# Miscellany

**Published:** 1902-02

**Authors:** 


### Miscellany.

Concealed Gold Bridge Attachment.—"A method of bridge attachment designed to take the place of a shell crown, an open-faced crown, or any other kind of crown, in the six anterior teeth, when entirely or comparatively free from caries."

This method is especially desirable in cases where all the central and lateral incisors are to be replaced, making the attachments to the cuspids. Devitalize the pulps, and fill the root-canals as usual. Bevel the lingual side of the tooth to be attached down to the incisive edge, allowing a liberal space for the thickness of the backing, to prevent the inferior incisors from impinging against it.

Take an impression of the prepared cuspid in either plaster or mouldine and pour in fusible metal. Using this as a die, swage the backing of No. 30 gauge, pure gold plate by driving the die into a soft pine block, cross-section. Make the backing large enough to cover the entire lingual aspect of the tooth and to overlap well the edges, particularly on the mesial side. This backing may or may not extend beneath the gum, as is deemed best by the operator. Through this backing insert a platinum or iridio-platinum wire, gauge about 15; place on the tooth and allow the post to extend well up into the canal. Fasten the post and backing together with Parr's wax, remove, invest and solder slightly together with 22- or 20-carat solder; replace on tooth and burnish perfectly, especially around the edges. Adjust dummies, wax together, remove and invest again, and flow with 20-carat solder. With a little finishing and reburnishing, the attachment is ready for use. In this class of attachments, as in gold inlay fillings, the edge adaptation and burnishing are very important, and must not be neglected. Well-nigh perfect edges may be obtained in this way, with proper manipulation of the pure gold backing. After the bridge is set reburnish all the edges.—H. M. Kirke, *Dental Digest.*

[It is a serious question whether "this method is especially desirable in cases where all the central and lateral incisors are to be replaced, making the attachments to the cuspids." The curvature of the arch produces a leverage when pressure is put upon the centrals that no attachment to the cuspids can withstand.—McClain.]

Soapstone.—Pulverized soapstone has several uses in the dental office. Have a quantity of the powder in a wide-mouthed bottle on the operating-case. A little rubbed on the dam where the holes are punched will make the application easy. The powder applied to the surface of cement fillings prevents its sticking to the instruments. In the laboratory, a pepper-box of the powder from which to sprinkle it on plaster after setting in the lower part of the flask sufficiently to handle makes a good separating medium. The powder sprinkled into the shoes, if a little tight, makes them feel larger.—John G. Harper, *Dental Register.*

---

To Prevent Unsoldering.—In cases where an investment is not indicated, it is frequently desirable to observe some precautions to avoid the unsoldering or re-fusing of parts previously united, which is usually accomplished by the mere presence of the investment itself when such is used. This may always be very easily prevented by coating or treating such surfaces with crocus (ferric hydrate), or a liquid solution of plumbago, or whiting in water or alcohol.—Hart J. Goslee, D.D.S., *Items of Interest.*

---

Cottonoid Strips with the Rubber Dam.—In using the rubber dam in the mouth of a patient, a cottonoid strip placed between the rubber and the lower lip is far superior to the napkin or muslin, and for the following reasons:

It adapts itself more readily to the anatomy of the parts, and hence lies in position without being held by a strap or, indeed, any precaution for retaining it in place.

It is soft, yielding, downy; consequently it has a more "kindly" feel to the tongue and lips.

It absorbs surplus saliva, and yet it does not need to be so frequently changed, if it is necessary to change it at all; therefore the annoyance of being compelled to stop our work in order to move a wet napkin along or replace it with a dry one is avoided. It supplants the saliva ejector in many instances, and it can be dispensed with altogether by substituting aseptic absorbent cotton, casting it aside as soon as it becomes full of saliva and renewing with fresh cotton.—Alphons Irwin, D.D.S., *Items of Interest.*

